# Complete Genome Sequence of *Pseudomonas* sp. Strain NC02, Isolated from Soil

**DOI:** 10.1128/genomeA.00033-18

**Published:** 2018-02-15

**Authors:** Joseph Cerra, Hailey Donohue, Alexander Kral, Molly Oser, Laura Rostkowski, Luke Zappia, Laura E. Williams

**Affiliations:** a Department of Biology, Providence College, Providence, Rhode Island, USA

## Abstract

We report here the complete genome sequence of *Pseudomonas* sp. strain NC02, isolated from soil in eastern Massachusetts. We assembled PacBio reads into a single closed contig with 132× mean coverage and then polished this contig using Illumina MiSeq reads, yielding a 6,890,566-bp sequence with 61.1% GC content.

## GENOME ANNOUNCEMENT

*Pseudomonas* is a diverse genus of the *Gammaproteobacteria* whose members are found in a variety of environments, including soil, water, and air (1). We report here the complete genome sequence of *Pseudomonas* sp. strain NC02, isolated from soil in eastern Massachusetts (42.0877, −71.23099). Williams and coworkers (2) are using NC02 to test the prey range of predatory bacteria (note that in that study, NC02 is listed as 0042). Genome information for NC02 will help us understand how predatory bacteria may be used for the biocontrol of pathogenic and antibiotic-resistant strains of *Pseudomonas*, which are considered a serious health care threat (3).

We extracted genomic DNA from 3 ml of overnight culture grown in Trypticase soy broth (TSB) at 30°C using the Wizard genomic DNA purification kit (Promega). Aliquots were used by the University of Maryland Institute for Genome Sciences to construct a PacBio library and by the University of Rhode Island Genomics and Sequencing Center to construct an Illumina library. Sequencing on a PacBio RS II instrument using P6-C4 chemistry yielded 110,769 subreads with an *N*_50_ value of 11,787 bp from one single-molecule real-time (SMRT) cell. For *de novo* assembly, we launched an Amazon EC2 instance of SMRT Portal version 2.3.0 and used Hierarchical Genome Assembly Process version 3 (HGAP3) (4), with an estimated genome size of 11 Mb and target coverage of 25×. This generated one 6,912,634-bp contig with 132× mean coverage. To circularize the contig, we used Gepard (5) to visualize the overlap between contig ends and BLAST (6) and EMBOSS extractseq (7) to specify coordinates and trim overlap, thereby generating a closed 6,890,354-bp contig.

To polish the closed contig, we processed 2 × 250-bp Illumina MiSeq reads using SolexaQA++ version 3.1.4 (8). We removed bases that had a quality score of <13 with DynamicTrim and then discarded reads that had <90 bp with LengthSort. This yielded 5,331,038 read pairs. Using the Burrows-Wheeler aligner “mem” (BWA-mem) algorithm version 0.7.13 (9), we mapped 98.8% of these reads to the closed contig. We sorted and indexed the alignment file with SAMtools (10) and then used Pilon version 1.22 (11) to identify and correct one single-nucleotide polymorphism (SNP) and 224 small indels, yielding a corrected 6,890,566-bp contig. To confirm this sequence, we used the same Illumina MiSeq reads and DynamicTrim quality score cutoff but adjusted the LengthSort cutoff to 85, 80, 75, 70, 60, or 50 bp, which gradually increased the total number of reads retained. When we aligned the read data sets resulting from each LengthSort cutoff against the corrected contig with BWA-mem, the same two indels (one single-base insertion and one single-base deletion) were identified. These were corrected by Pilon to generate the final genome sequence of 6,890,566 bp, with 61.1% GC content.

Annotation with the Prokaryotic Genome Annotation Pipeline (PGAP) predicted 6,255 protein-coding sequences, 903 of which are annotated as hypothetical proteins, along with 66 tRNAs and 5 rRNA operons. To attempt to further classify NC02, we aligned *rpoD* by BLASTN to the nonredundant GenBank database, which returned two top hits with 99% identity, *Pseudomonas yamanorum* (GenBank accession no. LT629793) and *Pseudomonas fluorescens* (GenBank accession no. CP012400). Because of the complexity of *P. fluorescens* taxonomy, we chose not to assign a species name.

### Accession number(s).

This complete genome sequence has been deposited in GenBank under the accession no. CP025624. The version described in this paper is the first version, CP025624.1.
